# Controlled freezing inactivates *Trichinella britovi* in wild boar meat: Insights from a murine infection model and multiplex polymerase chain reaction

**DOI:** 10.14202/vetworld.2025.1667-1674

**Published:** 2025-05-19

**Authors:** Olimpia C. Iacob, Aurelian-Sorin Paşca, Laura Andreea Olariu, Larisa Maria Ivănescu, Mihai Mareş

**Affiliations:** 1 Department of Parasitology and Parasitic Diseases, Faculty of Veterinary Medicine, “Ion Ionescu de la Brad” Iasi University of Life Sciences, 700490, Romania; 2 Department of Pathological Anatomy, Faculty of Veterinary Medicine, “Ion Ionescu de la Brad” Iasi University of Life Sciences, 700490, Romania; 3 Department of Public Health, Faculty of Veterinary Medicine, “Ion Ionescu de la Brad” Iasi University of Life Sciences, 700490, Romania

**Keywords:** controlled freezing, multiplex polymerase chain reaction, murine infection model, *Trichinella britovi* larvae, wild boar meat

## Abstract

**Background and Aim:**

Trichinellosis remains a public health concern globally due to the zoonotic potential of consuming undercooked meat infected with *Trichinella* spp. larvae. *Trichinella britovi*, known for its moderate freeze tolerance, presents a food safety challenge, particularly in game meat such as wild boar. This study aimed to evaluate the infectivity of *T. britovi* larvae in wild boar meat subjected to prolonged freezing under controlled conditions.

**Materials and Methods:**

Muscle samples (50 g each) from a wild boar naturally infected with *T. britovi* were frozen for 56 days at four temperatures: −18°C, −20°C, −29°C, and −40°C. Post-thaw, larval viability was assessed through artificial digestion, and infectivity was tested in a murine model using BALB/c mice. Each experimental group (n = 5 mice) received 120 larvae through gavage over 2 days. After 56 days, mice were euthanized, and muscle tissues were examined histologically. Molecular confirmation was performed using multiplex polymerase chain reaction on formalin-fixed tissues.

**Results:**

Despite larval motility post-thaw, no viable *T. britovi* DNA was detected in the muscle tissues of infected mice. Histological examination showed structures resembling *Trichinella* cysts in all experimental groups, but these were not molecularly confirmed. The control group remained negative throughout.

**Conclusion:**

Controlled freezing at temperatures as low as −18°C for 8 weeks rendered *T. britovi* larvae in wild boar meat non-infectious in a murine model. These findings suggest that freezing may be a viable strategy for reducing the risk of trichinellosis transmission through game meat. However, given species-specific variability and environmental influences, further studies across diverse conditions are warranted to refine food safety protocols.

## INTRODUCTION

Trichinellosis is a globally important parasitic zoonosis that arises from the consumption of raw or undercooked meat harboring infective larvae of the genus *Trichinella* [1]. The genus currently comprises ten recognized species – *Trichinella* *spiralis*, *Trichinella britovi*, *Trichinella nativa*, *Trichinella murrelli*, *Trichinella nelsoni*, *Trichinella papuae*, *Trichinella chanchalensis*, *Trichinella pseudospiralis*, *Trichinella zimbabwensis*, and *Trichinella patagoniensis* – as well as three genotypes: T6, T8, and T9 [2]. These species differ significantly in their biological traits, particularly in reproductive capacity and resistance to low temperatures, which are influenced by both intrinsic factors (e.g., parasite genotype and host physiology) and environmental variables [3].

In Europe, wildlife plays a critical role in maintaining the enzootic cycle of *Trichinella* spp., acting as reservoirs that facilitate long-term persistence and interspecies transmission within ecosystems [4]. Scavenger species that feed on decomposing carcasses represent the primary hosts maintaining this parasitic cycle [5]. Human infections typically occur through the ingestion of meat- or meat-derived products from infected domestic animals (e.g., pigs and horses) or wild animals (e.g., wild boars and bears) that are consumed raw or undercooked [6–8]. Notably, muscle larvae (ML) of *T. nativa*, genotype T6, *T. britovi*, and *T. spiralis* have demonstrated the ability to survive extended exposure to subzero temperatures in the muscle tissues of their natural hosts, underscoring their public health relevance [5, 7, 9].

*T. britovi* is widely distributed throughout the Palearctic region, from the −6°C January isotherm in the north to the northern and western regions of Africa [10]. It infects a broad range of wild mammals, including members of the Canidae, Felidae, Mustelidae, Ursidae, Viverridae, and Suidae families, across Europe, Asia, and Northwest Africa [5]. Human transmission is sporadic and typically associated with the consumption of meat from infected animals, including pork, horse, wild boar, or bear. Although *T. britovi* exhibits moderate infectivity and persistence within host muscle tissue [11], its tolerance to freezing renders it particularly problematic in the context of food safety. Freeze-tolerant *Trichinella* species can potentially remain infectious even after undergoing conventional commercial freezing processes for meat preservation [12].

The European Food Safety Authority has highlighted concerns regarding the inadequacy of the present freezing protocols to inactivate cold-resistant *Trichinella* species or genotypes in pork, emphasizing the need for species-specific experimental validation of time–temperature inactivation parameters [13]. In particular, empirical data on the freeze resistance of *T. britovi* in pork and wild boar meat are scarce [13]. Routine veterinary inspections, along with public health education regarding the handling and preparation of meat, are essential but insufficient alone to mitigate the risk. Many consumers continue to believe that domestic freezing practices ensure the safety of potentially infected meat [11].

Therefore, it is critical to recognize that the effectiveness of freezing as an inactivation method is influenced by multiple factors, including the *Trichinella* species or genotype present and the type of meat – whether pork, horse, or game [14].

Despite extensive investigations into the survival and infectivity of *Trichinella* spp. under freezing conditions, data on the cold resistance of *T. britovi* in wild boar meat remain limited and often inconclusive. Previous studies have produced contradictory results regarding the efficacy of freezing at conventional commercial temperatures (e.g., −18°C–−20°C) in rendering *T. britovi* larvae non-infectious. Most experimental models have focused on *T. spiralis* or used laboratory-infected pork rather than naturally infected wild game. In addition, the validation of larval inactivation has often relied on morphological assessments or digestion techniques without molecular confirmation. Given the growing consumer preference for wild game meat and the increasing recognition of *T. britovi* as a zoonotic threat in Europe and surrounding regions, there is a pressing need for rigorously controlled *in viv*o studies that combine histopathological and molecular approaches to assess larval viability following freezing treatments across a gradient of subzero temperatures.

This study aimed to evaluate the viability and infectivity of *T. britovi* ML in naturally infected wild boar meat subjected to controlled freezing at four distinct temperatures (−18°C, −20°C, −29°C, and −40°C) over a period of 8 weeks. Using a murine infection model, coupled with histological examination and multiplex polymerase chain reaction (PCR) for molecular confir-mation, this study sought to determine whether such freezing conditions are sufficient to inactivate *T. britovi* larvae and prevent subsequent transmission. This integrated experimental design not only addresses a critical gap in food safety research but also informs future guidelines on the safe handling and processing of wild boar meat in regions where *T. britovi* is endemic.

## MATERIALS AND METHODS

### Ethical approval

All procedures involving animals were carried out in accordance with Directive 2010/63/EU of the European Parliament and of the Council on the protection of animals used for scientific purposes (Official Journal of the European Union, September 22, 2010) and institutional guidelines from the “Ion Ionescu de la Brad” Iasi University of Life Sciences. Ethical approval for the study was obtained from the Faculty of Veterinary Medicine Iasi, Institutional Ethics Committee (protocol code no. 1941, December 4, 2022).

Throughout the experimental period, animal welfare standards regarding housing, nutrition, hydration, rest, and hygiene were strictly observed. Each group was monitored daily for clinical signs and behavioral changes, with stress-minimizing practices employed. Euthanasia was performed promptly as per the experimental design. This study adhered to the ARRIVE guidelines for reporting *in vivo* animal experiments.

### Study period and location

The study period was conducted from January 2023 to October 2024, in the Parasitic Diseases Clinic, in the Pathological Anatomy and Public Health laboratories of the Faculty of Veterinary Medicine, “Ion Ionescu de la Brad” Iasi University of Life Sciences, in the Veterinary Sanitary and Food Safety Laboratory Iasi and in the Parasitic Diseases Clinic of the Faculty of Veterinary Medicine, Cluj-Napoca, Romania.

### Study novelty and justification

To the best of our knowledge, this is the first experimental study in Romania to evaluate the post-freezing viability of *T. britovi* larvae isolated from wild boar meat, using a controlled murine model in conjunction with molecular confirmation through multiplex PCR. The study’s novelty lies in the use of four sub-zero temperature gradients (−18°C, −20°C, −29°C, and −40°C) sustained over an 8-week period, which exceeds the freezing durations typically assessed in prior literature.

The focus on wild boar meat, as opposed to pork, was based on identified knowledge gaps in *T. britovi* epidemiology in Romania. Additionally, wild boar is a known natural reservoir of *T. britovi*, and the consumption of game meat is increasing among consumers, thereby increasing the relevance of this research.

### Experimental animals

A total of 25 BALB/c (BAGG/Albino/C) mice, aged 35 days and weighing 25 ± 0.4 g, were used. The animals were divided into five groups, consisting of four experimental groups and one control group, each with five mice. Housing, feeding, and environmental conditions complied with the standards set by Directive 63/2010/EC.

### Infectious material collection and preparation

The infectious material consisted of *T. britovi* ML isolated from a wild boar hunted in the Vaslui region. Species identification was confirmed by multiplex PCR at the Institute of Public Veterinary Hygiene and Health, Bucharest. Larval density was assessed by direct trichinelloscopic examination and varied across tissues: diaphragm (10–52 cysts/field × 28 fields), intercostal muscles (3–7 cysts/field × 20 fields), tongue (1–5 cysts/field × 15 fields), cervical muscles (1–3 cysts/field × 12 fields), and dorsal muscles (1–2 cysts/field × 9 fields). Each field measured 0.8 × 0.3 mm. Four 50 g muscle samples from heavily parasitized tissues (diaphragm, intercostals, and tongue) were frozen at −18°C, −20°C, −29°C, and −40°C for 8 weeks (56 days).

### Thawing and viability evaluation

Frozen samples were thawed gradually over 24 h–12 h at +4°C and 12 h at room temperature (21°C). Each sample underwent artificial digestion according to Commission Implementing Regulation (EU) 2015/1375. Larvae were found to be alive post-digestion and were stored in saline for 24 h. The Euzeby method was used for counting (30 larvae/0.15 mL). A portion of the larvae was used for mouse infection, and the rest was preserved in 70% ethanol for molecular studies.

### Experimental infection design

Larvae were administered through oral gavage at 60 larvae/mouse per day for 2 consecutive days (120 larvae total). Experimental groups were infected as follows: batch 1 (−18°C), batch 2 (−20°C), batch 3 (−29°C), and batch 4 (−40°C). Batch 5 served as an uninfected control.

- Temperature Justification: The selected freezing temperatures reflect contradictory findings in the literature and represent the hypothesized safety thresholds for human meat consumption
- Freezing Duration: The 8-week freezing period was based on previous studies, safety regulations, and the cold tolerance of *Trichinella* larvae
- Larval Viability Assessment: Viability was confirmed microscopically by observing motile larvae immediately before infection
- Each group received larvae only from its designated frozen sample
- Standardization: All mice received identical larval doses determined by the Euzeby counting method to minimize variability
- Blinding and Randomization: Histopathological and molecular analyses were performed by blinded, independent researchers
- Control Validation: The control group remained uninfected throughout the study and was negative by multiplex PCR.

### Monitoring and necropsy

The experiment lasted 56 days, during which mice were monitored daily for clinical and behavioral changes. Upon completion of the study, mice were euthanized in accordance with Directive 2010/63/EC. A necropsy was performed, and five muscle samples (diaphragm, abdominal wall, tongue, forelimb, and dorsal) were collected from each mouse (125 total). Samples were fixed in 10% formalin, paraffin-embedded, sectioned at 5 μm, and stained with Masson’s trichrome. Microscopic analysis and imaging were performed using a Leica DM750 microscope, Leica ICC50 camera, 5MPX, LAS image acquisition software (Leica Application Suite) version 2018 (Leica Microsystems, Germany).

### Molecular confirmation through multiplex PCR

DNA samples were extracted from paraffin-embedded muscle tissue fragments using the method described by Pikor *et al*. [15]. Gene amplification was performed using the multiplex PCR method in the parasitology laboratory of the Faculty of Veterinary Medicine, Cluj-Napoca. The positive control *T. britovi* ML was provided by the Faculty of Veterinary Medicine Cluj-Napoca, courtesy of Professor Călin Gherman, PhD, from the discipline of Parasitology and Parasitic Diseases.

The identification of *T. britovi* ML was performed using the multiplex PCR method, as described in the protocol established by Pozio and La Rosa [16]. Two pairs of primers were used. Primer sets (for amplicon sizes): I: Ts 173; II: Tb 127; 253. Primer pair I: 5’-GTTCCATGTGAACAGCAGT-3’; 5’-CGAAAACATACGACAACTGC-3’. Primer pair II: 5’-GCTACATCCTTTTGATCTGTT-3’; 5’-AGACACAATATCAACCACAGTACA-3’ were designated to amplify the internal transcribed spacers (ITS)1 and ITS2 and the expansion segment V region of the ribosomal DNA. 10 μL of total DNA was subjected to multiplex PCR in a 30 μL mixture reaction. The mix for the detection of the target sequence contained 1× PCR buffer, 3 mM MgCl_2_, 0.2 mM of each deoxynucleotide triphosphate, 0.3 μM of each primer, and 1 U of Taq polymerase. Amplification was performed as follows: Initial denaturation at 95°C for 4 min; 40 cycles of 95°C for 10 s, 55°C for 30 s, and 72°C for 30 s; and a final extension cycle at 72°C for 3 min. DNA fragments were analyzed by electrophoresis in 2% agarose gel in 1× Tris-acetate-ethylenediaminetetraacetic acid (EDTA) buffer (40 mmol/L Tris–HCl, 2 mmol/L acetate, and 1 mmol/L EDTA) and stained with ethidium bromide. The bands in the gel were visualized and photographed under ultraviolet (UV) light.

### Significance of the experimental design

The present study is distinguished by its systematic evaluation of *T. britovi* cold tolerance across four controlled sub-zero temperature conditions using naturally infected wild boar meat. In contrast to earlier investigations that often focused on a single temperature or relied exclusively on morphological assessments, this research employed a comprehensive murine infection model, combined with molecular diagnostics through multiplex PCR, to assess both larval viability and infectivity following freezing. This integrated approach provides robust and novel evidence that enhances present risk assessments related to the microbiological safety of game meat in areas where *T. britovi* is endemic.

## RESULTS

### Histopathological findings

**Figure 1:**
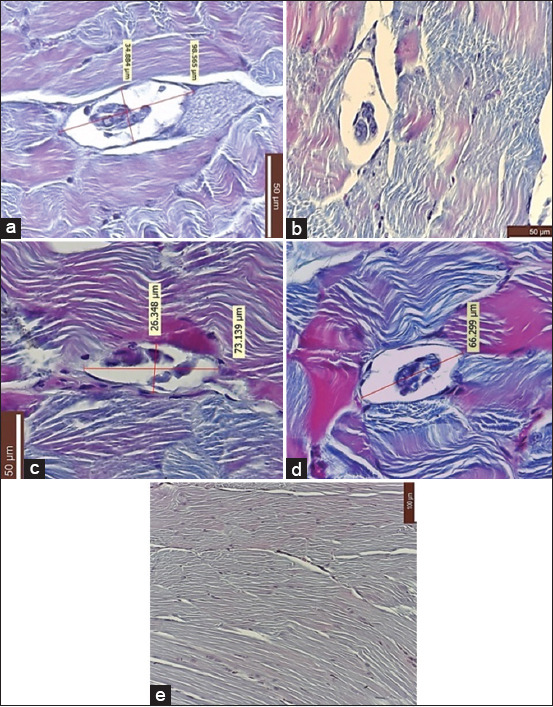
(a) Muscle tissue taken from mice experimentally infected with *Trichinella britovi* muscle larvae (ML) frozen controlled at −18°C (a: Batch 1); (b) muscle tissue taken from mice experimentally infected with *T. britovi* ML frozen controlled at −20°C (b: Batch 2); (c) muscle tissue taken from mice experimentally infected with *T. britovi* ML frozen controlled at −29°C (c: Batch 3); (d) muscle tissue taken from mice experimentally infected with *T. britovi* ML frozen controlled at −40°C (d: Batch 4); and (e) muscle tissue taken from mice of control batch (e: control batch). Muscle tissue samples were sectioned at 5 μm and stained using Masson’s trichrome technique 200×. In Figures a-d, fusiform cystic formations aligned along muscle fibers of uniform size are observed. Each cystic structure was enclosed by a wall and contained internal material, regardless of the temperature at which the wild boar meat was frozen, from which the infective larvae for the experimental batches (a–d) originated. In the control batch (e), cystic formation was absent.

Histopathological examination of muscle tissues from all infected batches revealed structures resembling *Trichinella* cysts, primarily localized within the abdominal musculature (Figure 1, panels a–d). These cyst-like structures were absent in the negative control group (Figure 1e).

### Molecular confirmation through multiplex PCR

**Figure 2:**
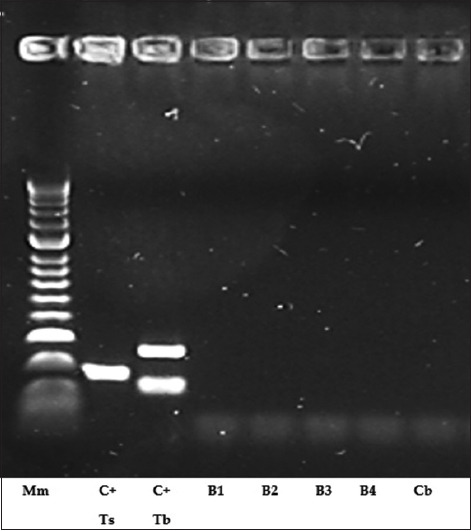
Ethidium bromide-stained 2.5% agarose gel under ultraviolet illumination demonstrating multiplex polymerase chain reaction amplification of *Trichinella britovi* muscle larvae. Captation: Mm (molecular marker); C+Ts (Control+ *Trichinella spiralis* (Zsolt); C+Tb (Control+ *T. britovi* (Zsolt); B1 (Batch 1); B2 (Batch 2); B3 (Batch 3); B4 (Batch 4); and cb (Negative control batch).

Multiplex PCR analysis confirmed the absence of *T. britovi* DNA in the muscle tissues of all experimentally infected mice and the control group (Table 1). Electrophoresis of ethidium bromide-stained 2.5% agarose gel under UV light demonstrated the PCR amplification profile of *T. britovi* ML (Figure 2).

**Table 1:** Results of the multiplex PCR test performed on batches of mice experimentally infected with *Trichinella britovi* ML frozen for 8 weeks at controlled temperatures.

Exp. batch/mice (n = 5)	Larval age	T (°C) freezing of wild boar meat	Freezing period (weeks)	Alive larve after thawing	Inf./120 larvae/mouse (weeks)	Histologically similar *Trichinella* cysts	PCR analysis of *Trichinella britovi*
1	Unknown	−18°C	8	Yes	8	Yes	Negative
2	Unknown	−20°C	8	Yes	8	Yes	Negative
3	Unknown	−29°C	8	Yes	8	Yes	Negative
4	Unknown	−40°C	8	Yes	8	Yes	Negative
Control batch	Control batch	Control batch	Control batch	Control batch	Control batch	Control batch	*Control + *T. britovi*

* For the PCR test, the positive control *T. britovi* larvae was provided by the Faculty of Veterinary Medicine Cluj-Napoca, courtesy of Professor Călin Gherman PhD from the discipline of Parasitology and Parasitic Diseases. PCR=Polymerase chain reaction, ML=Muscle larvae, Exp. batch=Experimental batch, T=Temperature, Inf.=Infection

## DISCUSSION

### Freezing resistance of *Trichinella* larvae in meat

Numerous studies have reported by Pozio *et al*. [17], Capó and Despommier [18], and Malakauskas and Kapel [19] the survival of *Trichinella* ML in frozen meat, highlighting implications for food safety. Freezing is a widely adopted method for preserving meat in both domestic and industrial settings. Several *Trichinella* species, including *T. nativa*, *T. britovi*, *T. spiralis*, and genotype T6, exhibit tolerance to freezing conditions [18, 20, 21].

This study presents the first experimental evaluation conducted in Romania, assessing the cold resistance of *T. britovi* ML in naturally infected wild boar meat. *T. britovi* (T3), distributed across temperate and Palearctic regions, parasitizes a broad range of domestic and wild mammals, and is characterized by moderate infectivity and cold tolerance [18].

### Larval viability post-freezing

Previous studies have indicated that freezing muscle tissue from wild game may not reliably inactivate *Trichinella* larvae, partly due to the presence of antifreeze proteins that protect larvae from ice crystal damage. These proteins enable larvae to persist in frozen carcasses until they are ingested by new hosts. Some species, such as *T. nativa* and T6, remain infective for several days at freezing temperatures even outside host tissue [18].

In the present study, larvae remained viable after freezing at −18°C, −20°C, −29°C, and −40°C for 8 weeks (Table 1). Following experimental infection in mice, histopathology revealed spindle-shaped structures resembling *Trichinella* cysts in the abdominal muscles. However, PCR analysis did not confirm the presence of *T. britovi* DNA. These results may reflect the low susceptibility of the mouse model to *T. britovi*, resulting in insufficient larval localization and encystment, or they may represent histological artifacts despite morphological similarities to cysts.

### Limitations of molecular detection

As noted by Franssen *et al*. [22], the sensitivity of PCR for detecting single *T. britovi* larvae may be reduced due to DNA degradation caused by deep freezing. In addition, repeated freeze–thaw cycles can impair DNA integrity through thermal shock [23]. In this study, samples were thawed only once, minimizing this risk. Despite potential limitations, multiplex PCR remains a highly sensitive and specific tool capable of identifying individual *Trichinella* larvae to the species and genotype level [24, 25].

### Comparison with existing literature

Previous studies by Pozio *et al*. [17], Gari-Toussaint *et al*. [26], and Kapel *et al*. [27] have yielded inconsistent findings on *T. britovi*’s viability post-freezing (Table 2). Pozio *et al*. [17] demonstrated larval survival at −20°C for 3 weeks in wild boar meat, while Gari-Toussaint *et al*. [26] found viability at −35°C for 1 week. Conversely, Kapel *et al*. [27] observed no infectivity in *T. britovi* larvae frozen at −18°C for 1–4 weeks in experimentally infected wild boars or pigs. Larval death in pigs may result from the absence of antifreeze proteins [18].

**Table 2:** Infectivity of *T. britovi* larvae in frozen naturally or experimentally infected pork or wild boar meat [12].

The origin of the infected pig	Larval age	Temperature (°C)	Weeks frozen	Infectivity of larvae after thawing	Reference
Naturally infected wild boar	Unknown	−20	3	Yes	[17]
Naturally infected wild boar	Unknown	−20	4	No	[17]
Naturally infected wild boar	Unknown	−35	1	Yes	[26]
Experimentally infected pigs	5–10 weeks	−18	1–4	No	[27]
Experimentally infected pigs	5–10 weeks	−5	1–4	Yes	[27]
Experimentally infected wild boar	5–10 weeks	−18	1–4	No	[27]
Experimentally infected wild boar	5–10 weeks	−5	1–4	Yes	[27]
Naturally infected pigs	Unknown	−18	1	No	––

The tolerance of *T. britovi* to freezing varies depending on host species, infection duration, and freeze–thaw protocols [12]. Malakauskas and Kapel [19] studied nine *Trichinella* isolates in rat muscle tissues and found that *T. nativa* alone remained infective after freezing at −18°C. Most encapsulated species (excluding *T. nelsoni*) survived at −5°C, while *T. pseudospiralis* endured for only 1 week. Larval age influenced survival: 10–20-week-old larvae tolerated freezing better than early or late-stage larvae. In the present study, larval age was unknown, though the 8-week freezing period aligns with favorable survival durations.

Hill *et al*. [28] reported that freezing protocols effective for inactivating *T. spiralis* were also adequate for *T. nativa*, *T. murrelli*, T6, and *T. pseudospiralis* in pork. These findings suggest that tailored freezing protocols may be necessary to ensure the safety of meat potentially infected with cold-resistant *Trichinella* species, including *T. britovi*.

### Implications for food safety and regulation

The persistent ambiguity regarding *T. britovi*’s freezing resistance necessitates careful evaluation, especially given its regular detection in both domestic and wild pigs in Europe. Relying solely on freezing as a mitigation strategy may not be sufficient. Products originating from regions endemic for *T. britovi* should not be considered safe based on freezing alone.

Consequently, reliable detection methods should be implemented, and consumer awareness regarding the limitations of home freezing must be reinforced. Further studies are warranted to assess the cold tolerance of *T. britovi* across different hosts and processing conditions, which will be instrumental in shaping effective food safety policies and international meat trade standards.

## CONCLUSION

This study demonstrated that controlled freezing of wild boar meat infected with *T. britovi* at temperatures of −18°C, −20°C, −29°C, and −40°C for 8 weeks effectively rendered the larvae non-infectious, as confirmed by the absence of detectable *T. britovi* DNA in experimentally infected mice using multiplex PCR. Although viable larvae were observed post-thaw under light microscopy, histological examination revealed structures resembling *Trichinella* cysts. However, molecular diagnostics confirmed the absence of true infection, suggesting inactivation of larval infectivity.

The findings support the potential utility of deep freezing as a practical intervention to mitigate the risk of *T. britovi* transmission through wild boar meat, particularly in endemic regions where game meat is commonly consumed. This research contributes to evidence-based recommendations for food safety practices and reinforces the importance of integrating molecular tools for reliable post-freezing assessment of parasitic viability.

A major strength of this study lies in its comprehensive experimental design, which combined a gradient of freezing temperatures with a murine infection model, histopathological analysis, and multiplex PCR confirmation. This multifaceted approach enabled a robust evaluation of larval viability beyond traditional morphological observations.

Despite the rigorous design, the study was limited by the use of a single host species (BALB/c mice), which may differ in susceptibility from other potential hosts, including humans. In addition, only one strain of *T. britovi* from a specific geographical region was examined, which may limit generalizability to other isolates or host-parasite interactions.

Further research should investigate the infectivity of *T. britovi* across various host species and meat matrices (e.g., pork and horse), as well as the effects of repeated freeze–thaw cycles and varying larval ages. Comparative studies with other *Trichinella* species under similar conditions will also be essential to establish standardized inactivation protocols suitable for regulatory adoption.

This study highlights the significance of temperature-duration parameters in assessing the safety of game meat that may be infected with cold-tolerant parasites. While freezing under controlled conditions appears promising for inactivating *T. britovi*, it should not replace routine meat inspection and molecular surveillance. These findings contribute valuable data to the scientific basis for food safety policy development and public health protection against trichinellosis.

## AUTHORS’ CONTRIBUTIONS

OCI and MM: Conceived and designed the study and drafted and revised the manuscript. OCI, ASP, LAO, LMI, and MM: Performed the experimental work. OCI, ASP, LAO, and LMI: Analyzed and interpreted the data. All authors have read, reviewed, and approved the final manuscript.
